# Nano@lignocellulose intercalated montmorillonite as adsorbent for effective Mn(II) removal from aqueous solution

**DOI:** 10.1038/s41598-018-29210-2

**Published:** 2018-07-18

**Authors:** Yuhong An, Xiaotao Zhang, Ximing Wang, Zhangjing Chen, Xiangwen Wu

**Affiliations:** 1 College of Material Science and Art Design, Inner Mongolia Agricultural University, Hohhot, 010018 P.R. China; 2 College of Science, Inner Mongolia Agricultural University, Hohhot, 010018 P.R. China; 3 Department of Sustainable Biomaterials Virginia Tech University, Blacksburg, VA 24061 USA; 4 College student village officials of Xieji village Xieji town Shanxian Country Shandong province, Heze, 274300 P.R. China

## Abstract

This paper describes the preparation of nano@lignocellulose (nano@LC) and a nano@lignocellulose/montmorillonite (nano@LC/MT) nanocomposite, as well as the capacity of the nano@LC/MT for adsorbing manganese ions from aqueous solution. The structure of nano@LC and nano@LC/MT was characterised by Fourier-transform infrared spectroscopy, X-ray diffraction, Scanning electron microscopy, and Transmission electron microscopy, which revealed that the diffraction peak of montmorillonite almost disappeared, infrared bands of the functional groups shifted, and morphology of the material changed after the formation of the composite. The optimum conditions for the adsorption of Mn(II) on the nano@LC/MT nanocomposite were investigated in detail by changing the initial Mn(II) concentration, pH, adsorption temperature, and time. The results revealed that the adsorption capacity of the nano@LC/MT nanocomposite for Mn(II) reached 628.0503 mg/g at a Mn(II) initial concentration of 900 mg/L, solution pH 5.8, adsorption temperature 55 °C, and adsorption time 160 min. Adsorption kinetics experiments revealed good agreement between the experimental data and the pseudo-second order kinetic model. The experimental data was satisfactorily fitted to the Langmuir isotherm. Adsorption-desorption results showed that nano@LC/MT exhibited excellent reusability. The adsorption mechanism was investigated through FT-IR and EDX spectroscopic analyses. The results suggested that nano@LC/MT have great potential in removing Mn(II) from water.

## Introduction

The heavy metal content in wastewater has sharply increased in the wake of modern industrialisation on account of mining, smelting, washing away of chemical fertilisers, industrial waste gas discharging, etc^1–4^. Heavy-metal pollutants are posing a potential threat to flora and fauna; furthermore, large quantities of toxic metal ions will eventually accumulate in the human body by way of the human food chain and can become extremely difficult to remove^5–7^. Manganese plays a significant role in biological growth^8^, and a Mn concentration limit of < 0.05 mg/L in drinking water has been stipulated by the World Health Organisation. Excessive intake poses a threat to our overall wellbeing^9,10^. We seem to overlook or neglect the toxicity of Mn and its potential to cause harm. The toxicity triggered by Mn leads to lesions and complex symptoms; for instance, the death of plant cells and degradation of cell components^11^, resulting in muscular trembling, fatigue, stimulation, or reduced equilibrium^12^. Manganese may also lead to Parkinson’s disease.

Dealing with the pollution of heavy metals is an urgent problem. Until now, a number of methods have been applied to solve this problem, such as chemical precipitation^13^, ion exchange^14^, membrane separation^15^, electro-remediation methods and flocculation^16^. However, most of these methods are not sufficiently efficient and environmentally benign in removing heavy-metal pollutants. Adsorption is an environmentally friendly and low-cost strategy to treat wastewater effectively, and natural polymeric materials have been chosen as first-rank raw adsorbent materials^17^.

Lignocellulose (LC) is an ideal biological adsorbent material owing to its recyclability, relative cheapness, and particular structural characteristics^18^. The major components of LC are cellulose, hemicellulose and lignin, providing LC with a variety of reactive functional groups, e.g., hydroxyl, phenolic, acetyl, methyl, and carboxyl moieties. Blending these constituents affords a stable three-dimensional structure rich in active sites for the adsorption of Mn ions^19,20^. However, the particular structure of LC hampers its reaction with other materials^21,22^. In general, mechanical methods^23–26^ are used to reduce the molecular weight of LC and release more functional groups for participation in composite formation reactions.

**Figure 1:**
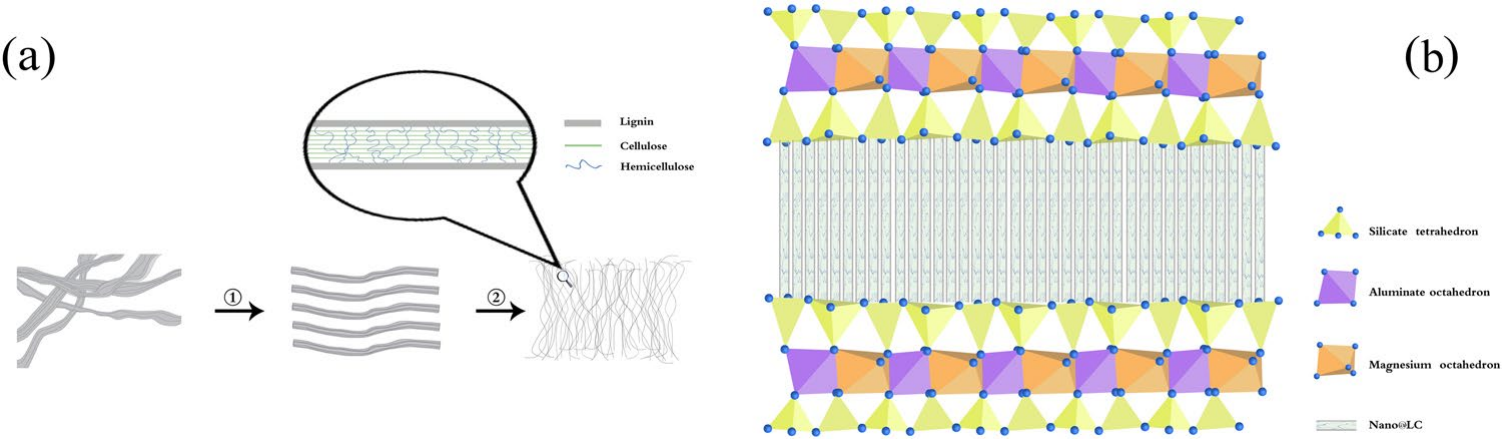
(**a**) Preparation of nano@LC: ① the intertwined lignocellulosic clusters are unwound and ② the LC beams are destroyed to afford nano@LC; (**b**) Schematic representation of the nano@LC/MT nanocomposite.

Clay materials have been typically used as cheap and easily obtained adsorbents in recent years. Among them, montmorillonite (MT) presents a mineral nanolamellar structure with high cation exchange capacity and high surface area^27,28^. However, the adsorption capacity of MT is not high enough for large-scale applications. In order to increase their adsorption capacity for Mn(II) cations^29^, MT was reacted with nano@LC to form a nanocomposite adsorbent by attaching the adsorbed functional groups of nano@LC to the structure of MT. Figure 1a shows the preparation process for nano@LC and Fig. 1b shows the structure diagram of the nano@LC/MT nanocomposite.

## Results and Discussion

### Characterization of the prepared materials

N_2_-adsorption/desorption isotherms provide qualitative information regarding the porosity of adsorbents. The textural parameters of MT and nano@LC/MT obtained from the N_2_-adsorption/desorption (V-Sorb 2800TP, Beijing GAPP Ltd.) isotherm are summarized in Table 1. From the results, the highest surface area (701.80 m^2^/g) and total pore volume (0.987 cm^3^/g) of nano@LC/MT were calculated using the *t*-plot method, respectively. According to the International Union of Pure and Applied Chemistry (IUPAC), the mesopore structure of nano@LC/MT can be supported by its average pore diameter (D_*p*_ = 6.165 nm), implying that most of the mesoporous was developed, with many pores generated on the surface of nano@LC/MT. Additionally, it also can be seen from the Table 1 that carbon and oxygen are the main components of nano@LC/MT (S-4800, Hitachi), because carboxyl and hydroxyl groups are potential functional groups for heavy metals. These signals were consistent with the FTIR spectral data. Based on the abovementioned discussion, nano@LC/MT can allow the formation of adequate activated sites and functional groups for chelation and complexation with heavy metals.

**Table 1:** Porosity structure parameters, elemental analysis and surface characteristics of the sample studied in this work.

Sample	S_*BET*_ (m^2^/g)	S_*langmuir*_ (m^2^/g)	V_*tot*_ (cm^3^/g)	V_*meso*_ (cm^3^/g)	V_*mic*_ (cm^3^/g)	D_*p*_ (nm)	C (at.%)	O (at.%)	H (at.%)	C/O (%)	C/H (%)	Zeta potential (mV)
MT	87.6	137.52	1.058	0.030	0.024	204.05	17.64	30.15	1.87	58.50	9.43	−24.07
nano@LC/MT	532.74	701.80	0.987	0.583	0.249	6.165	32.90	47.08	3.47	69.88	9.48	−39.49

Number of analyses: three. S_*BET*_—specific surface area; S_*ext*_—mesopore surface area, V_*tot*_—total pore volume, V_*meso*_—mesopore volume,V_*mic*_—micropore volume, D_*meso*_—average mesopore size, D_*mic*_—average micropore size, D_*p*_—average pore size.

**Figure 2:**
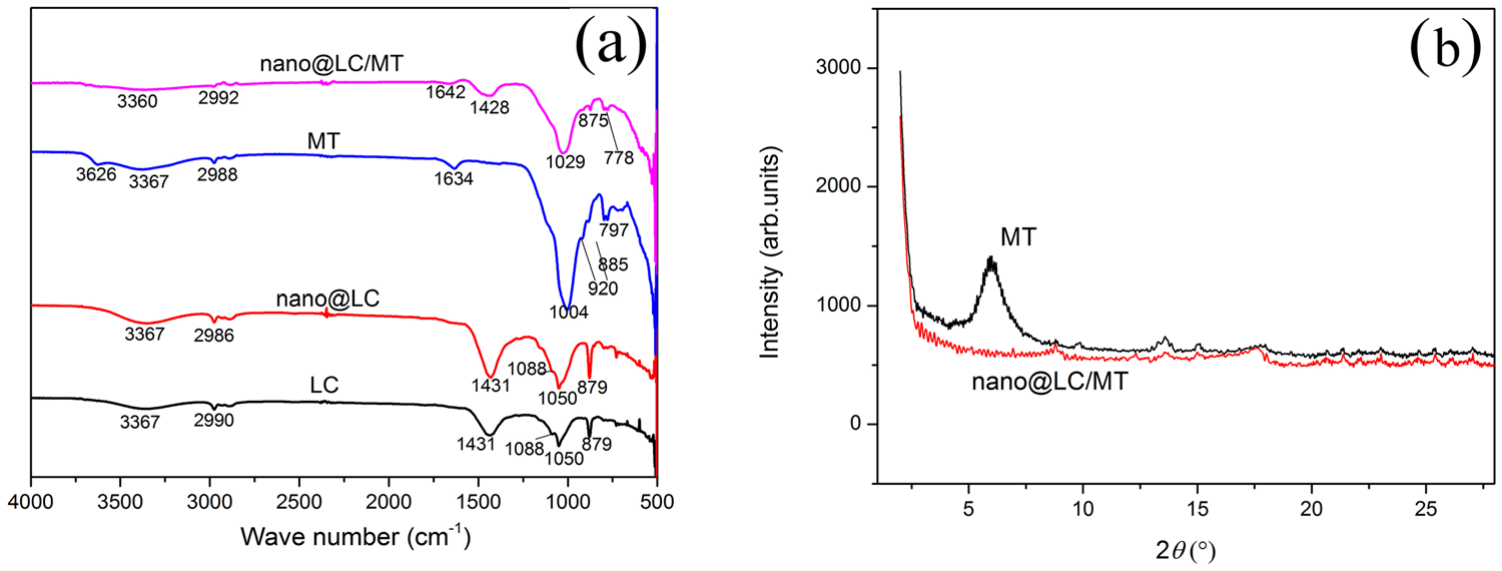
(**a**) FT-IR spectra of LC, nano@LC, MT and the nano@LC/MT. (**b**) XRD powder patterns of MT and the nano@LC/MT.

FT-IR spectroscopy (Spectrum 65, Perkin Elmer) allows monitoring the functional groups of materials and their changes. Figure 2a shows the infrared absorption spectra of LC, nano@LC, MT and nano@LC/MT. The infrared absorption band position is very similar upon comparing LC with nano@LC, but the absorption band intensity in nano@LC is significantly enhanced. LC and nano@LC reveal an absorbance bands at 3367 cm^−1^ attributed to acidic -OH groups and the hydroxyl groups involved in water–water hydrogen bonding^30^. The bands at 2986 cm^−1^ and 2990 cm^−1^ are assigned to the C-H stretching on methyl and methylene in LC and nano@LC, respectively. The band at 1088 cm^−1^ is assigned to the C-O stretching in nano@LC^31^. The bands at 1431 cm^−1^ and 1050 cm^−1^ are assigned to C=C bond stretching and O-H (C-OH) bond bending^30,32^. The absorption band at 879 cm^−1^ corresponds to aromatic and phenol C-H stretching bonding. It can be concluded that the nano-crystallisation process did not change the molecular structure of LC upon comparing the spectra in LC and nano@LC; however, the intensity of the infrared spectrum absorption band is significantly larger in nano@LC, which may indicate that the bundled LC was separated into a finer size after treatment in the ultrasonic wave cell pulveriser.

In order to confirm the framework and functional groups of MT, its spectrum was recorded and exhibited bands at 3626 cm^−1^ and 1004 cm^−1^ attributed to the stretching vibrations of the structural O-H groups and the siloxane -(SiO)_*n*_- stretching vibration^33^. The bands at 2988 cm^−1^ assigned to the -OH bending vibration of H_2_O of MT. The bands at 885 cm^−1^ and 920 cm^−1^ correspond to the in-plane vibrations of AlMgOH and AlAlOH, respectively^34^. The FT-IR spectrum of MT displays a band at 797 cm^−1^ due to Si-O tetrahedral bonding^35^. The band attributed to crystal water^36^ bending is centred at 1634 cm^−1^. By comparing MT and nano@LC/MT, the bands at 3626 cm^−1^ and 920 cm^−1^ in nano@LC/MT have disappeared. These observations prove that the structural O-H groups of MT react with nano@LC, and that the AlAlOH and AlMgOH moieties are destroyed during the reaction. The bands at 2992 cm^−1^ assigned to the -OH bending vibration of H_2_O of nano@LC/MT. The hydrogen atoms on acid moieties are removed and afford a symmetric COO^−^ stretching vibration band at 1428 cm^−1^. The intensity of the band at 885 cm^−1^ shifts to 875 cm^−1^, while the intensity of the 797 cm^−1^ band weakens and shifts to 778 cm^−1^, indicating the alteration of the Si-O tetrahedral structure in MT upon formation of the composite. Nano@LC enters the layers of MT and the C-O moieties possibly react with the broken -(SiO)_*n*_- bonds to form Si-O-C bonds, as suggested by the band at 1029 cm^−1^ in nano@LC/MT, resulting in the disappearance of the Si-O band at 1004 cm^−1^. It can be concluded from the information of FT-IR spectra that -OH, Si-O, Al-O and C-O, -COO^−^, -C=O groups were involved in the intercalation process.

The changes in the lamellar spacing between MT and the nano@LC/MT were determined by X-ray diffraction (XRD-6000, SHIMADZU CORPORATION). Figure 2b shows the XRD patterns of MT and the nano@LC/MT nanocomposite. The characteristic diffraction peak of MT is observed at 5.92° (*d* = 1.49 nm), which showed typical nanostructure features. However, the typical diffraction peak for MT practically disappears in the nano@LC/MT, the XRD patterns indicated that nano@LC/MT had been intercalated into an MT interplayer. According to the XRD and FT-IR analyses, it can be concluded that nano@LC molecules have intercalated the MT layer, upon which the nano@LC -OH, C=C, C-H, -COO^−^, -C=O and other active groups undergo a series of coordination and complexation reactions with the -OH, Si-O, Al-O and other groups of MT to afford the nano@LC/MT nanocomposite.

**Figure 3:**
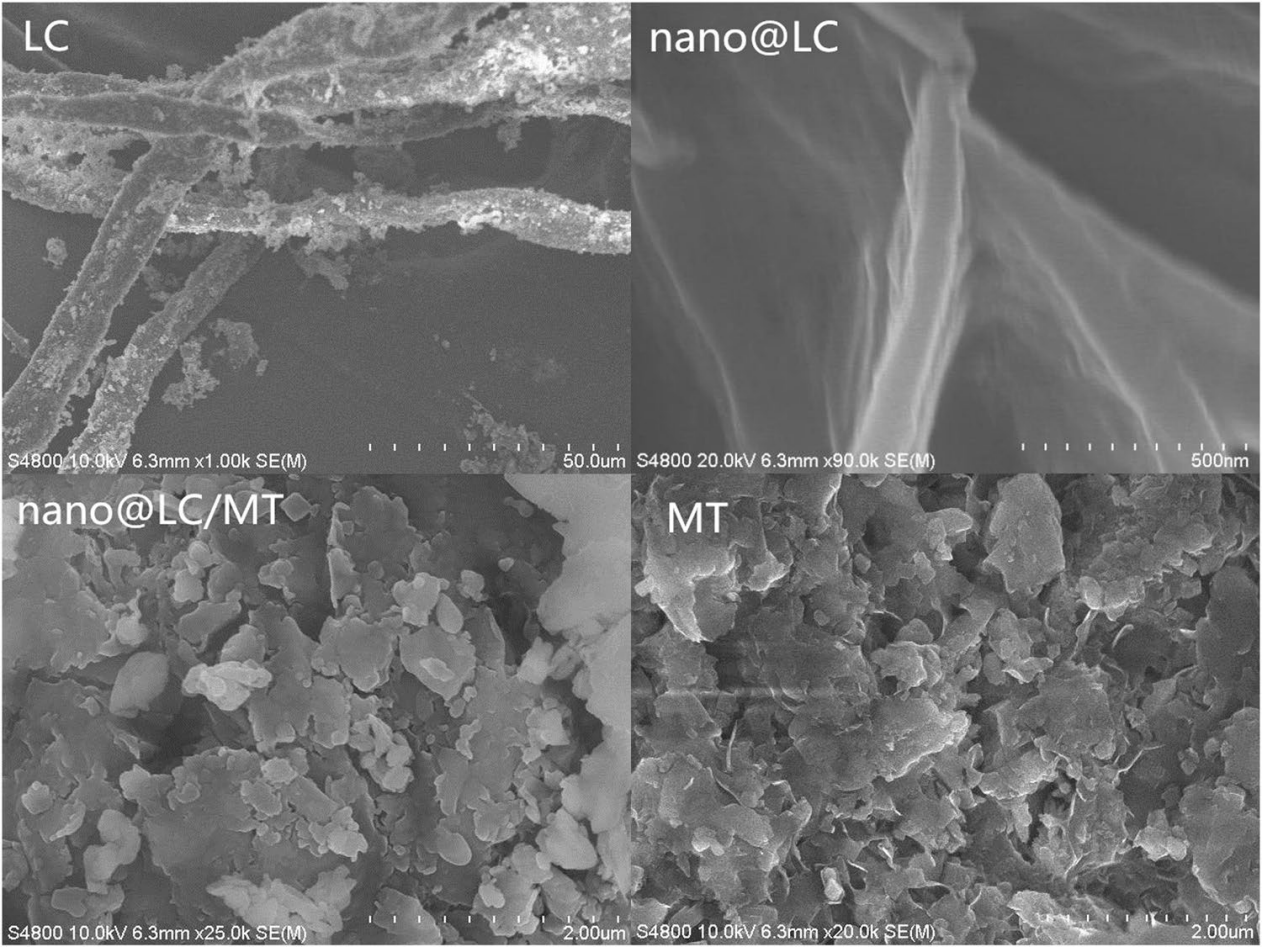
SEM images of LC, nano@LC, MT and the nano@LC/MT.

The surface morphology of LC, nano@LC, MT and the nano@LC/MT nanocomposite were observed by SEM (S-4800, Hitachi) (Fig. 3). A pre-sonicated lignocellulosic diameter of about 4‒13 µm is observed in LC; however, a sonicated lignocellulosic diameter of about 70‒100 nm is revealed in nano@LC. The LC clusters are separated into smaller sizes after treatment with an ultrasonic wave cell pulveriser (SM-1200D, Shunma Ltd., Nanjing) and the wound structure is broken down to form more dispersed nano@LC. It also can be seen that the MT clay particles are finely divided into a sheet structure with a closely arranged morphology. Many tiny interstices exist between closely connected sheets, which are the channels via nano@LC fully contacts the MT structure. The micro-morphology of nano@LC/MT is shown that the laminated sheet structure becomes loose and the interstices between the sheets are filled by nano@LC. This indicates that the MT crystalline phase is destroyed upon incorporation of nano@LC, which is effectively dispersed within the nanoscale sheets of MT.

**Figure 4:**
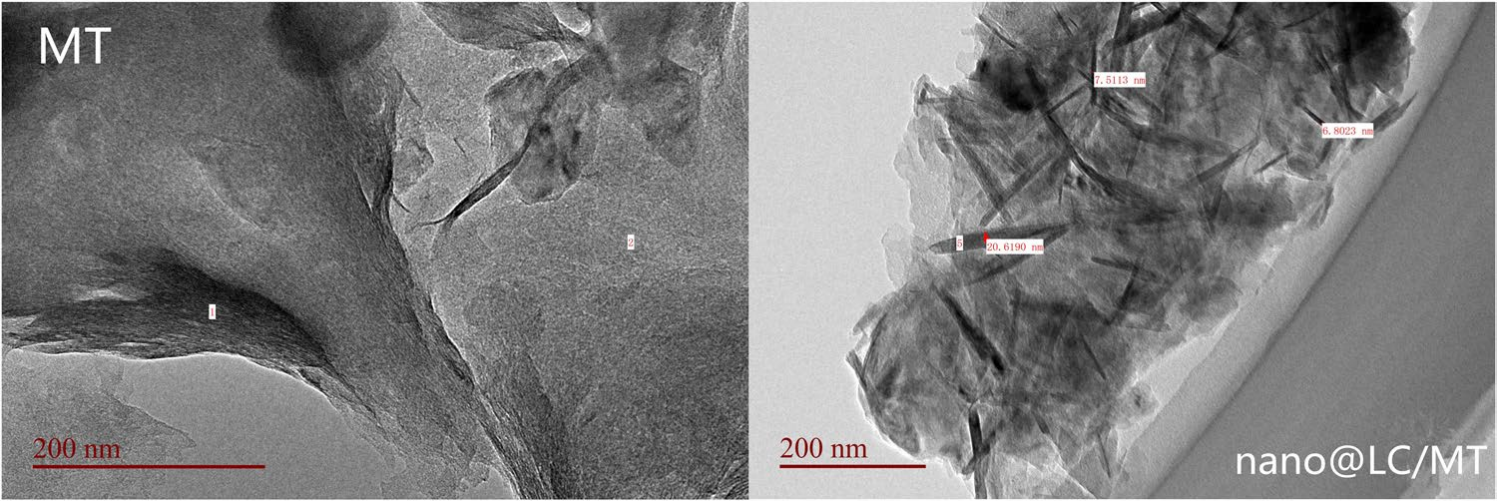
TEM images of MT and the nano@LC/MT nanocomposite.

Figure 4 shows the TEM (JEM-2100, JEOL Ltd.) images of purified MT and nano@LC/MT. The close-knit and overlapping structural morphology of MT is shown in Fig. 4. As it can be seen the structural morphology of the nanocomposite becomes loose and the overlapped structure breaks as MT reacts with nano@LC. At the same time, many fine rod-like structures appear in the morphology of the nano@LC/MT nanocomposite. Thus, the XRD patterns and TEM images indicate that nano@LC was successfully inserted into the lamellae of MT, after which its crystalline structure was destroyed.

## Adsorption Studies

### Effect of mass ratios of nano@LC and MT

**Figure 5:**
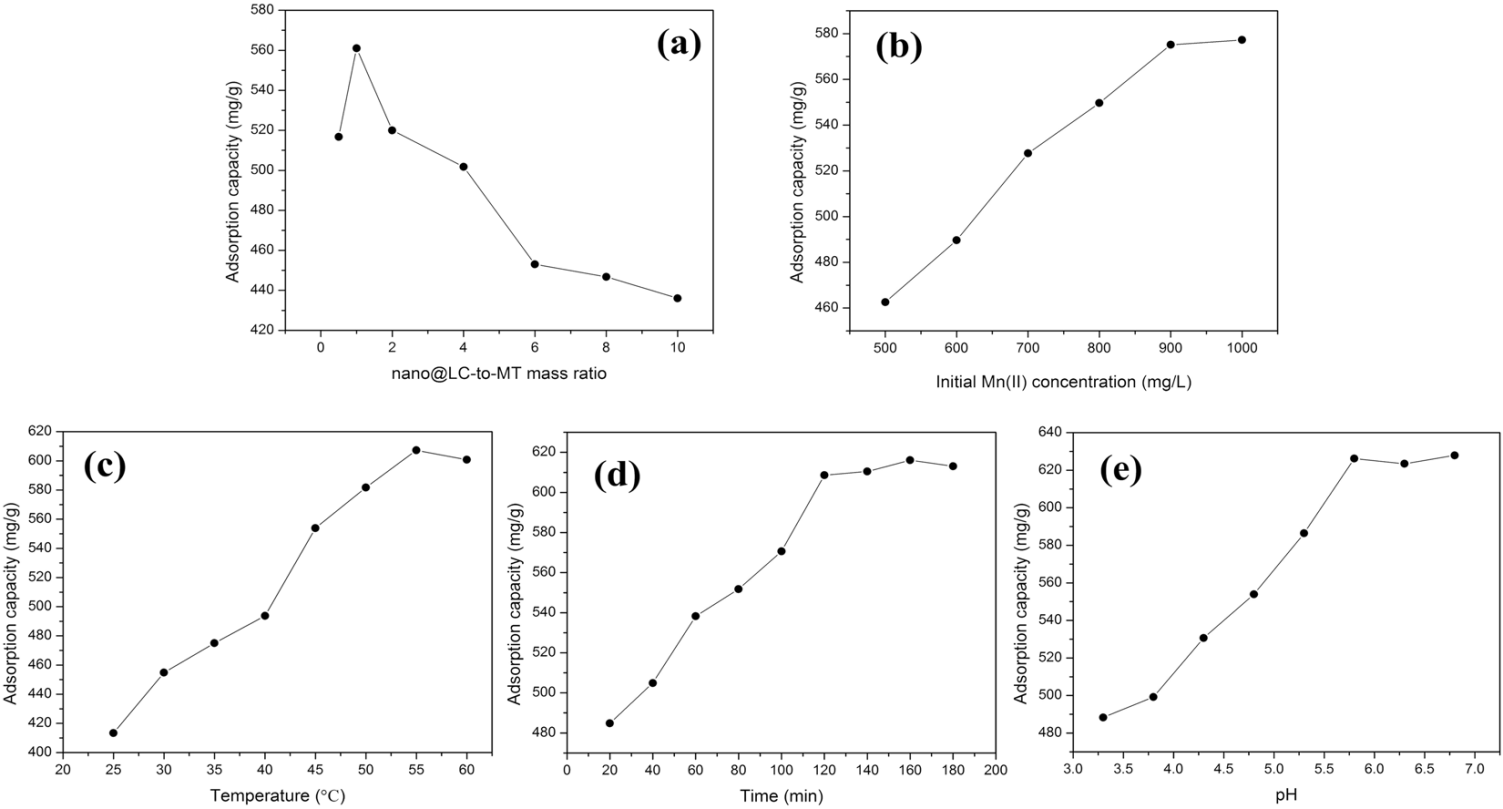
Effect of various factors on the adsorption capacity on the adsorption capacity of the nano@LC/MT. (**a**) Mass ratio of nano@LC-to-MT; (**b**) Initial Mn(II) concentration; (**c**) Temperature; (**d**) Time; (**e**) pH.

As the mass ratios of nano@LC to MT increases, nano@LC/MT mixtures tend to shrink and agglomerate, which can facilitate the separation of adsorbents from aqueous solution. Figure 5a shows the effect of mass ratios of nano@LC to MT on the adsorption capacity of the nano@LC/MT. Adsorption conditions: sample dose, 0.0500 g; Mn(II) concentration, 900 mg/L; pH, 5.8; temperature, 55 °C; time, 160 min. As seen from Fig. 5a, the adsorption capacity of Mn(II) increase with increasing of mass ratios of nano@LC to MT, but the adsorption capacity apparently decrease when the mass ratio of nano@LC to MT exceed 1:1. It may be explained by the following two reasons: on the one hand, the amounts of active hydroxyl, carboxyl groups increase with increasing of the mass ratio of nano@LC to MT, which result in an increase in adsorption capacities of Mn(II). On the other, the introduction of nano@LC in the MT galleries can improve the flocculation capacity of MT and increase adsorption capacity for Mn(II). However, the adsorption capacities reduce sharply when the mass ratio of nano@LC to MT exceeds 1:1, which is attributed to the amount of intercalated nano@LC is saturated.

### Effect of the initial Mn(II) concentration

The initial Mn(II) concentration was found to be the main driving force for adsorptive transportation. As shown in Fig. 5b, the adsorption capacity of the nano@LC/MT varied with the initial concentration of the Mn(II) solution. Adsorption conditions: sample dose, 0.0500 g; the mass ratios of nano@LC to MT, 1:1; pH, 5.8; temperature, 55 °C; time, 160 min. The adsorption capacity of the nano@LC/MT gradually increased with the initial Mn(II) concentration until reaching equilibrium. The above results suggest that the initial concentration increases the adsorption ability, but further increasing the initial concentration has no or weak influence on the adsorption capacity once the nano@LC/MT adsorption sites become saturated.

### Effect of the temperature

Figure 5c shows the effect of adsorption temperature on the adsorption capacity of the nano@LC/MT. Adsorption conditions: sample dose, 0.0500 g; the mass ratios of nano@LC to MT, 1:1; Mn(II) concentration, 900 mg/L; pH, 5.8; time, 160 min. The plot shows that increasing the temperature is beneficial for the adsorption properties of the nano@LC/MT; however, the adsorption capacity of the nano@LC/MT begins to decline at temperatures higher than 55 °C. The reason behind these phenomena is the synergistic effect of physical adsorption and chemisorption, in which the chemisorption process absorbs heat and the physical adsorption process releases heat. Therefore, the adsorption capacity exhibits a downward trend when the temperature increases above 55 °C.

### Effect of time

The effects of different adsorption times on the adsorption capacity of the nano@LC/MT towards Mn(II) ions are shown in Fig. 5d. Adsorption conditions: sample dose, 0.0500 g; the mass ratios of nano@LC to MT, 1:1; Mn(II) concentration, 900 mg/L; pH, 5.8; temperature, 55 °C. The adsorption capacity of the nano@LC/MT gradually increases, then slows down, and eventually reaches the equilibrium state with time. This is because the functional groups of the nanocomposite gradually react with the Mn(II) ions and the adsorption sites become gradually occupied. The adsorption capacity of the nanocomposite reaches its limit under the current adsorption conditions when the adsorption sites and functional groups are close to saturation.

### Effect of pH value

The pH value is an important controlling parameter in the adsorption process of Mn(II) ions. Surface characteristics of MT and nano@LC/MT (SOE-070, DelsaNano C, BeckmanCoulter) are listed in Table 1. The Table 1 results evidence that intercalation reaction induced a larger negative magnitude of zeta potential, a greater contant of acidic groups present on the surface of nano@LC/MT, that upon dissociation gives rise to a more negatively charged surface, which generated a greater potential to bind positively charged Mn(II) in solution. Figure 5e shows the effect of pH value on adsorption capacity. Adsorption conditions: sample dose, 0.0500 g; the mass ratios of nano@LC to MT, 1:1; Mn(II) concentration, 900 mg/L; temperature, 55 °C; time, 160 min. It not only affects the adsorption sites on the adsorbent surface but also the chemical properties of Mn(II) ions in an aqueous solution^37^. The adsorption capacity of the nano@LC/MT increases with pH in the acidic range 3.3–6.8. At low pH values (pH < 3.8), Mn(II) sorption was almost negligible. This behavior may be due to strong electrostatic repulsion between the positively charged surface of nano@LC/MT and Mn(II), almost hindering the metal-binding process. A significant increase in adsorption takes place for 3.8 < pH < 5.8, as the pH values of solution increases, repulsion interactions seem to be reduced and the extent of Mn(II) sorption increases, presumably due to an ion exchange mechanism between H^+^ of the surface and Mn(II). As the pH increased, the anion group concentration (-COO^−^, -OH^−^, etc.) increased, and the coordination and chelation ability of Mn(II) with nano@LC/MT gradually increased. However, when the pH was higher than 5.8, Mn(II) could react with a basic pH regulator, which resulted in facile complexation or precipitation and therefore affected the determination in adsorption capacity. It was determined that the optimum pH for adsorption was 5.8.

### Adsorption kinetics studies

**Figure 6:**
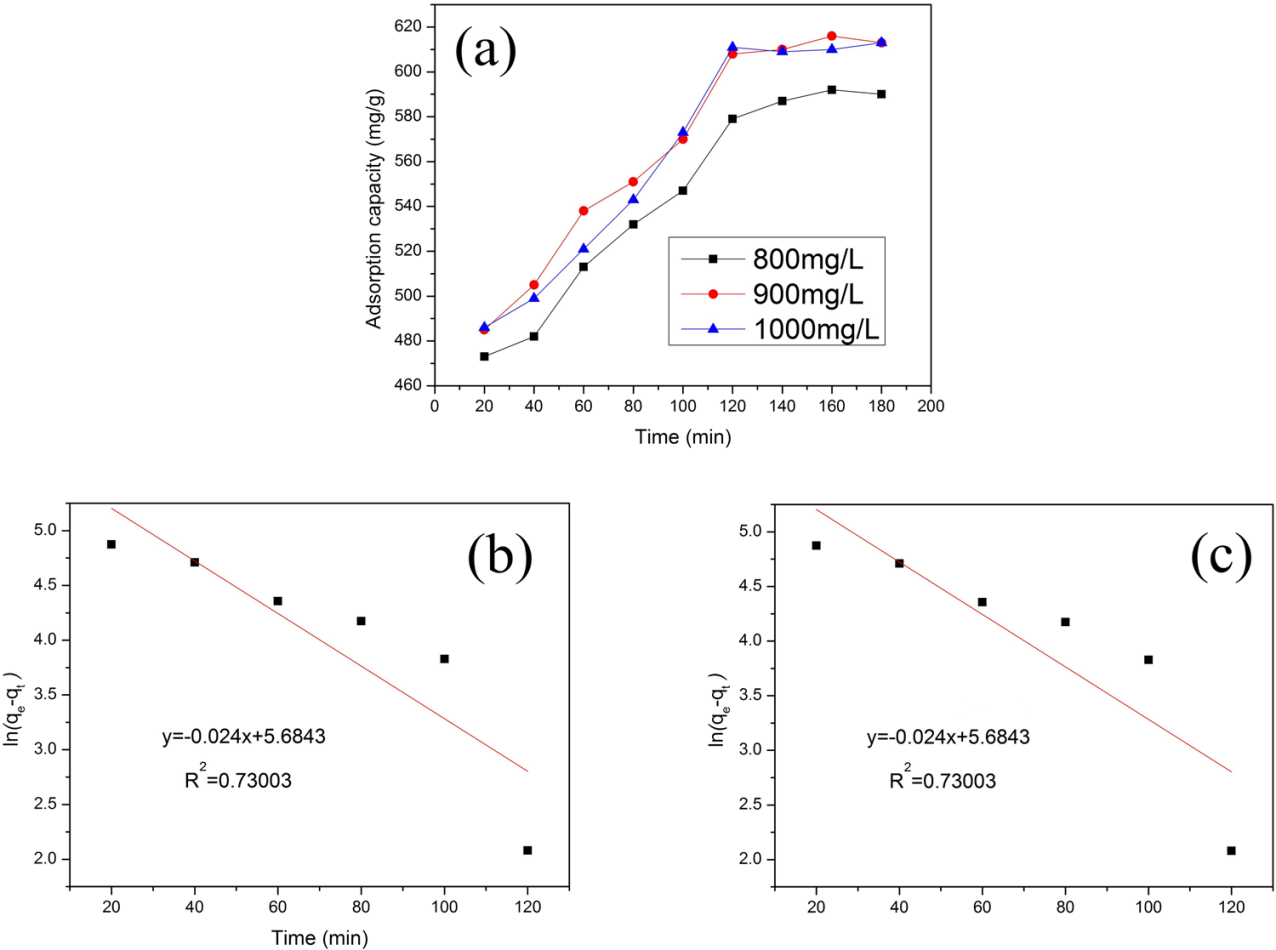
(**a**) Effect of time on the adsorption by the nano@LC/MT nanocomposite at various initial Mn(II) concentrations. (**b**) Pseudo-first order and (**c**) pseudo-second order adsorption kinetic curves of the experimental data. (Adsorption experiments-sample dosage, 0.05 g; the mass ratios of nano@LC to MT, 1:1; initial Mn(II) concentrationinitial, 900 mg/L; pH value, 5.8; temperature: 55 °C).

In order to confirm that the adsorption process is time-dependent, initial concentrations of Mn(II) ions were selected as 800, 900 and 1000 mg/L, and the adsorption capacity of the nano@LC/MT and adsorption equilibrium time were used to infer the adsorption kinetic model for the adsorption of Mn(II) ions on the nanocomposite (Fig. 6a). To analyse the Mn(II) adsorption kinetics on the nanocomposite, pseudo-first order and pseudo-second order equations were used^38^. Equations (1) and (2) are the pseudo-first order and pseudo-second order kinetic equations, respectively.

1 $$\mathrm{ln}({q}_{e}-{q}_{t})=\,\mathrm{ln}\,{q}_{e}-{k}_{1}t$$

The plot of ln (*q*_e_ − *q*_*t*_) versus *t* gives a straight line with slope −*k*_1_ and intercept ln *q*_e_.

2 $$\frac{1}{{q}_{t}}=\frac{1}{{k}_{2}{{q}_{e}}^{2}}+\frac{t}{{q}_{e}}$$

The plot of *t*/*q*_*t*_ versus *t* gives a straight line with a slope of 1/*q*_e_ and intercept of 1/*k*_2_*q*_e_^2^. Using the value of *q*_e_ calculated from the slope, the value of *k*_2_ is determined from the intercept.

In those equations, *q*_e_(mg/g) is the adsorption capacity at equilibrium, *q*_*t*_(mg/g) is the adsorption capacity at time *t*, and *k*_1_ (min^−1^) and *k*_2_ [g·(mg/min)^−1^] are the rate constants of the pseudo-first and pseudo-second order kinetic equations, respectively.

The results of the adsorption kinetics and linear fitting models are shown in Table 2 and Fig. 6b,c. For the adsorption of Mn(II) ions by the nano@LC/MT, the linear fitting correlation coefficient (*R*^2^) of the pseudo-second order adsorption kinetic equation is higher than that of the pseudo-first order kinetic equation. Therefore, it is obvious that chemical adsorption should be the rate limiting step of the adsorption of Mn(II) onto the nano@LC/MT.

**Table 2:** Kinetic parameters for Mn(II) adsorption on nano@LC/MT.

Metal	Parameter	Pseudo-First-Order		Pseudo-Second-Order	
Mn(II)	*R* ^2^	0.73003		0.99106	
	Constants	*k*_1_	0.02400 min^−1^	*k*_2_	0.00015 min^−1^
		*q*_*ec*_	249.2118 mg/g	*q*_*ec*_	641.0526 mg/g
		*q*_*e*_	628.0503 mg/g	*q*_*e*_	628.0503 mg/g

### Adsorption isotherm studies

**Figure 7:**
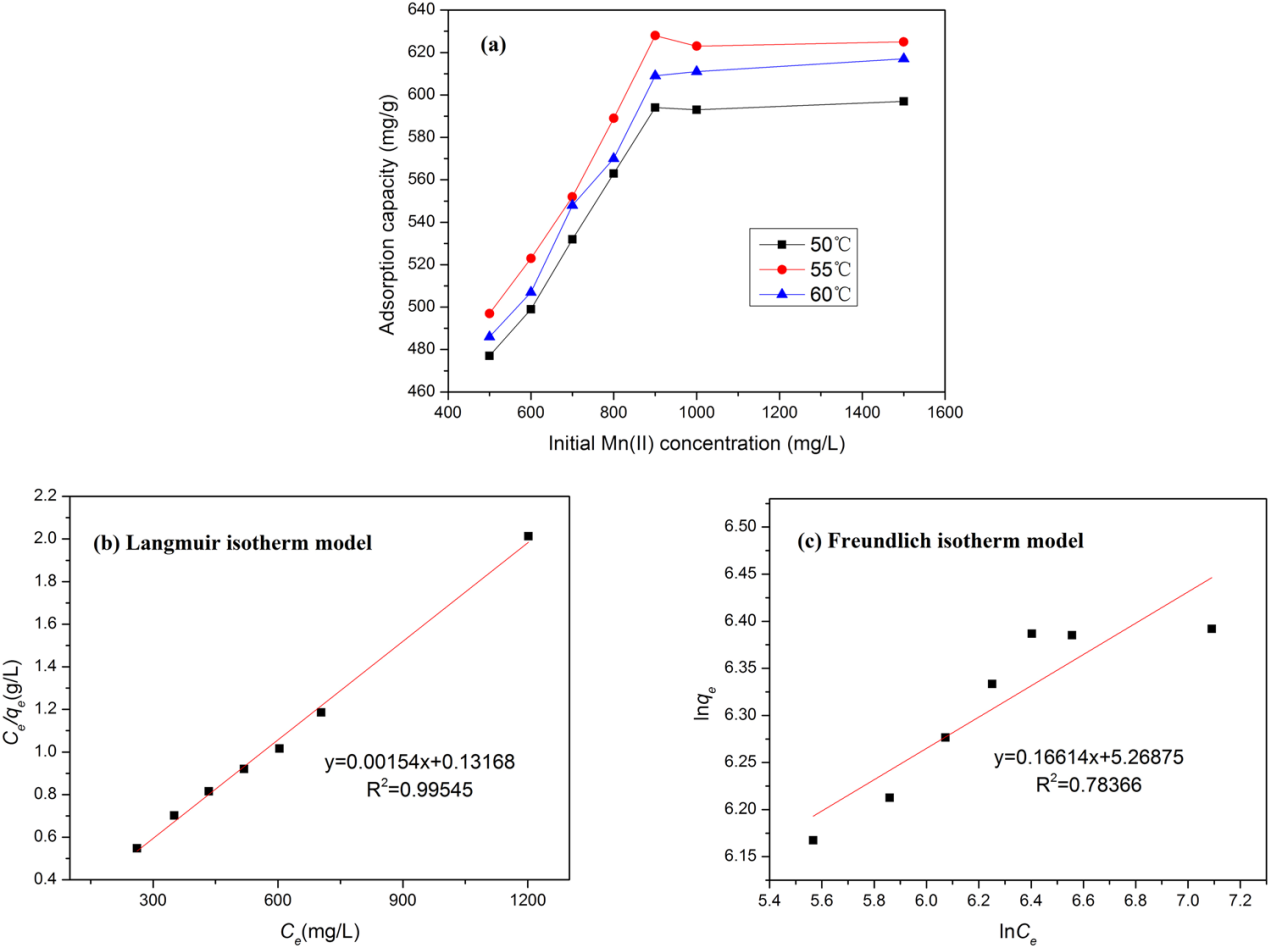
(**a**) Effect of the initial Mn(II) concentration on the adsorption by the nano@LC/MT at various temperatures; (**b**) Langmuir and (**c**) Freundlich isotherm curves of the experimental data. (Adsorption experiments-sample dosage, 0.05 g; the mass ratios of nano@LC to MT, 1:1; pH value, 5.8; temperature: 55 °C; time, 480 min).

Mn(II) ion solutions were prepared at initial concentrations of 500, 600, 700, 800, 900, 1000, and 1500 mg/L. Figure 7a shows the adsorption capacity of the nano@LC/MT at different initial Mn(II) concentrations and adsorption temperatures of 50, 55, and 60 °C.

Experiments were carried out under optimal adsorption conditions. The adsorption equilibrium data were analysed according to the Langmuir and Freundlich isotherm models Equations (3) and (4).

3 $$\frac{{C}_{e}}{{q}_{e}}=(\frac{1}{b{q}_{m}})+(\frac{{C}_{e}}{{q}_{m}})$$

4 $$\mathrm{ln}\,{q}_{e}=\,\mathrm{ln}\,{K}_{f}+\frac{1}{n}\,\mathrm{ln}\,{C}_{e}$$

where *q*_e_ (mg/g) and *C*_e_ (mg/L) are the adsorption capacity and concentration of Mn(II) ions at equilibrium, respectively; *q*_*m*_ (mg/g) and *b* (L/mg) are the Langmuir constants; and *K*_*f*_ (L/g) and *n* (L/mg) are the Freundlich constants.

Table 3 shows the Langmuir and Freundlich isotherm model parameters. The linear correlation coefficient (*R*^2^ = 0.9883) of the Langmuir isotherm adsorption model is greater than that of the Freundlich isotherm adsorption model (*R*^2^ = 0.9009). This indicates that the mechanism likely involves single-layer adsorption. The *q*_max_ values of Mn(II) on the nano@LC/MT nanocomposite were compared with those of other adsorbents and are listed in Table 4. It can be concluded that the *q*_max_ values of other materials were much lower than those of the nano@LC/MT. Consequently, the high adsorption capacity in this paper revealed that the nano@LC/MT can be employed as an excellent novel adsorbent to remove Mn(II) from aqueous solution.

**Table 3:** Langmuir and Freundlich isotherm model parameters.

Metal	Parameters	Langmuir		Freundlich	
Mn(II)	*R* ^2^	0.9883		0.9009	
	Constants	*b*	0.0083 L/mg	*K*_*f*_	116.4323 L/g
		*q*_max_	628.0503 mg/g	*n*	3.9079 L/mg

**Table 4:** *q*_max_ value for the adsorption of Mn(II) on different adsorbents.

Adsorbents	*q*_max_ (mg/g)	Reference
nano@LC/MT nanocomposite	628.0503	This paper
PSA–GO	165.5	^40^
Crab shell particles	69.90	^41^
Maize stalks	16.61	^42^
PVA/CS hydrogels	10.52	^43^
Acid-Treated Corncob Biomass	7.87	^44^
PLDC	7.00	^45^
Birbira (Melita Ferrginea) leaves activated carbon	3.41	^3^

### Desorption and reusability studies

The regeneration of nano@LC/MT is one of the key factors for its economic viability in practical applications. Reusability studies were conducted to regenerate heavy metals loaded on nano@LC/MT. If the strong acids, for example HCl can desorb Mn(II) ions, it can be concluded that the attachment of the Mn(II) onto the nano@LC/MT is by ion exchange or electrostatic attraction. Therefore, HCl at a concentration of 0.06 mol/L was selected as the eluent for desorption of Mn(II) from nano@LC/MT. Table 5 shows the adsorption/desorption results for Mn(II) and the regeneration of nano@LC/MT for five consecutive cycles. The reusability results suggest that the adsorption capacities for Mn(II) was almost unaffected, even after four cycles. This trend might be responsible for the negligible amount of materials lost during the regeneration cycles. In summary, nano@LC/MT can be used repeatedly for four cycles with little loss of initial adsorption capacity and possesses good regenerative properties towards Mn(II) ions in solutions.

**Table 5:** Nano@LC/MT adsorption/desorption capacities for Mn(II) in five consecutive cycles.

Cycle Number	1st	2nd	3rd	4th	5th
Adsorption *q*_e_ (mg/g)	628.05	602.17	586.65	568.29	202.47
Desorption *q*_e_ (mg/g)	598.33	595.83	571.02	553.24	150.81

(Adsorption experiments-sample dosage, 0.05 g; the mass ratios of nano@LC to MT, 1:1; initial Mn(II) concentration, 900 mg/L; pH value, 5.8; temperature: 55 °C; time, 160 min. Desorption experiments-sample dosage, 0.05 g; HCl concentration, 0.06 mol/L; desorption temperature: 55 °C; desorption time, 60 min).

### Adsorption Mechanism

**Figure 8:**
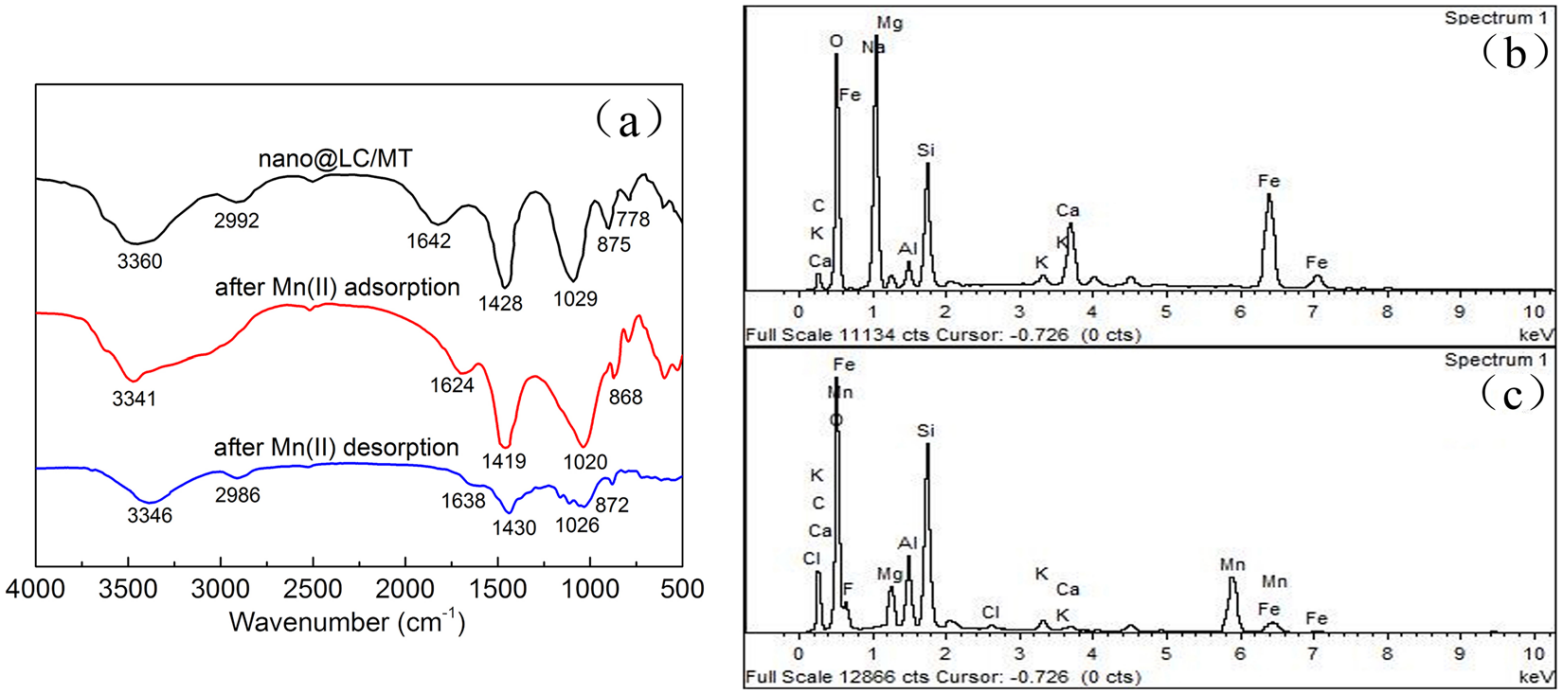
(**a**) FT-IR spectra of nano@LC/MT, after adsorption Mn(II) and after desorption Mn(II); (**b**) EDS results for the nano@LC/MT nanocomposite before and after the adsorption of Mn(II) ions.

FT-IR spectra of nano@LC/MT, after adsorption and desorption Mn(II) are shown in Fig. 8a. The adsorption bands at 3360 cm^−1^ in nano@LC/MT are attributed to the intramolecular O-H stretching vibration absorption peak, as well as the characteristic absorption band of intermolecular hydrogen bonding between phenol and alcohol molecules. The band shifted to a lower wavenumber at 3441 cm^−1^ after the adsorption of Mn(II), indicating that some of the O-H and corresponding hydrogen bonds interacted with Mn(II), and this band weakened after desorption. The characteristic adsorption band at 1642 cm^−1^ in nano@LC/MT, corresponding to the asymmetric stretch vibration of the C=O bond in carboxylic acids, decreased after adsorption of Mn(II). It appeared again after desorption at a lower wavelength 1638 cm^−1^. The vibration absorption peak of the carboxyl O-H bond, located at 1428 cm^−1^ in nano@LC/MT, reduced after Mn(II) adsorption, shifting at 1430 cm^−1^ after desorption. Moreover, the absorption band at 875 cm^−1^ in nano@LC/MT, which represents the stretching vibration absorption of the aromatic and phenol C-H stretching vibration, moved to a lower wavelength after absorption, and then shifted back down to 872 cm^−1^ after desorption. Based on the above-mentioned results, it was tentatively concluded that protons of the hydroxyl and carboxyl functional groups of nano@LC/MT were replaced by Mn(II) and the free carboxyl groups became carboxylates after adsorption. Generally, ion exchange occurred, and chemical bonds were formed between Mn(II) and the -OH and -COO^−^ groups of nano@LC/MT. Moreover, slight changes were observed in the FT-IR spectra of Mn(II)-loaded-nano@LC/MT, and they were basically restored to their original shape after desorption, which signaling that it is a highly-efficient renewable adsorbent.

EDS (S-4800, Hitachi) was used to determine the content of Mn(II) in the nano@LC/MT after the adsorption experiments. As shown in Fig. 8b, the EDS results for the nano@LC/MT revealed a content of C (32.90%), O (47.08%), Si (10.77%), Al (2.82%), and Mg (2.37%). The EDS spectrum of the Mn(II)-loaded-nano@LC/MT is shown in Fig. 8c. The two peaks corresponding to Mn(II) suggest a content of 10.88%. Combined adsorption isotherm, and adsorption kinetic studies were also carried out to evaluate the adsorption capacity of the nano@LC/MT towards Mn(II). The conclusion illustrated the interaction between the corresponding functional activated groups and Mn(II) ions, which was further confirmed by the following FT-IR analysis of the adsorption mechanisms.

## Conclusion

In summary, this paper reports the preparation of nano@LC and a nano@LC/MT nanocomposite, whose adsorption capacity towards Mn(II) ions was evaluated. The characterization data confirmed the successful preparation of nano@LC and the nano@LC/MT nanocomposite. The nano@LC/MT was effectively used for the removal of Mn(II) ions from aqueous solutions via adsorption. Under optimal conditions (sample dose = 0.0500 g; the mass ratios of nano@LC to MT = 1:1; initial Mn(II) concentration = 900 mg/L, Temperature = 55 °C, pH = 5.8, time* = *160 min), the maximum adsorption capacity of the nano@LC/MT for Mn(II) ions reached 628.0503 mg/g. In addition, the adsorption kinetics and isotherms were satisfactorily fitted to the pseudo-second order kinetic equation (*R*^2^ = 0.9911) and Langmuir isotherm model (*R*^2^ = 0.9883), respectively. The experimental results showed that the adsorption equilibrium was mainly influenced by single-layer chemisorption phenomena. The adsorption/desorption experiments demonstrated that the adsorption and desorption capacity of nano@LC/MT remained at a relatively high level after four rounds of adsorption/desorption recycling. The FT-IR and EDS results revealed that the adsorption mechanism was mainly by chemical interactions between Mn(II) and nano@LC/MT. This study showed that the nano@LC/MT nanocomposite could be used as an excellent and renewable potential adsorbent for the removal of Mn(II) ions from aqueous solutions.

## Materials and Methods

### Materials

LC was purchased from Beijing Huaduo Biotech, Ltd. (China) and MT (CEC = 100 meq/100 g) from Zhejiang Feng Hong Clay Chemical Co. (China). MT was washed with deionised water to remove any impurities, dried at 85 °C, milled, and sieved to afford a final size of 200 mesh. The molecular weight of MnCl_2_·4H_2_O (Tianjin Fengchuan Chemical Reagent Co., China) is 197.91 g/mol. All the other chemical reagents used were of analytical grade and the solutions were prepared using distilled water.

### Nano@LC

LC (0.50 g) was added to a 250 mL beaker, to which 250.00 mL of a 20 wt% sodium hydroxide solution was added. The mixture was stirred with a glass rod to obtain a homodispersion. An ultrasonic amplitude transformer bar was added to the mixture above, and the suspension was treated on an ultrasonic-wave cell pulveriser (SM-1200D, Shunma Ltd., Nanjing) for 150 min at 1080 W. Centrifugation of the suspension after ultrasonic processing afforded the desired material (nano@LC).

### nano@LC/MT nanocomposite

A 25 wt% sodium hydroxide solution (15 mL) was added to an Erlenmeyer flask (50.00 mL). Then, nano@LC was added to the flask and stirred for 30 min at 50 °C. MT (0.50 g) was ladled to the beaker (50.00 mL) and stirred for 30 min at room temperature (23 °C–27 °C). Subsequently, MT was mixed with nano@LC and the mixture was placed on a temperature-controlled magnetic stirrer for 4 h at 50 °C. The product was washed to pH 7.0 with deionised water, dried for 4 h at 85 °C (DZF-6210, Shanghai Yiheng Scientific Instrument Co., Ltd. Shanghai), and pulverised into a powder that was sieved to 200-mesh size.

### Adsorption Experiment

The nano@LC/MT nanocomposite (0.05 g) was precisely weighed, then added to an Mn(II) liquor (100 mL, pH value 5.8). The solution was mixed in a thermostatic shaker (TED, Taisite Ltd., Tianjin) at 60 °C for 120 min at a constant speed of 120 rpm. Adsorption experiments were performed by varying the mass ratios of nano@LC to MT, the initial Mn(II) concentration, pH, adsorption temperature, and adsorption time. The influence of pH value on Mn(II) removal was studied by adjusting the pH of the Mn(II) solution to different values (3.3, 3.8, 4.3, 4.8, 5.3, 5.8, 6.3, and 6.8) with a 0.1 mol/L HCl or NaOH solution. Upon completion of the adsorption process, the mixture was centrifuged at 5000 rpm for 5 min. The Mn(II) concentration of the supernatant was determined by the potassium periodate method^39^. The absorbance of Mn(II) complex using potassium periodate was measured at 525 nm on a double beam UV-visible spectrophotometer (TU-1901, Beijing Purkinje General Instrument Co., Ltd. China). The concentration of the Mn(II) solution was determined by a linear regression equation (*y* = = 0.0447*x* −0.0012, *R*^2^ = 0.9997), which was used to determine the adsorbed amount of manganese on the nano@LC/MT. Taking into account the experimental error and averaged values, three independent replicates confirmed the Mn(II) removal experiments to be reproducible under the same conditions. The amount of metal adsorption at equilibrium (*q*_*t*_) was calculated according to Eq. (5):

5 $${q}_{t,1}=\frac{({C}_{i}-{C}_{t,1})\times {V}_{1}}{{m}_{1}}$$

where *q*_t,1_ (mg/g) is the capacity of adsorption at time *t* (min); *C*_i_ and *C*_t,1_ (mg/L) are the Mn(II) initial concentration and concentration at time *t* (min), respectively; *V*_1_ (L) is the volume of the Mn(II) ion solution; and *m*_1_ (g) is the mass of adsorbent.

### Desorption and regeneration experiments

The Mn(II)-loaded-nano@LC/MT (0.05 g) was accurately weighted, transferred into 50 mL HCl, and put into an ultrasonic cleaning machine (KS-300EI, Qindao Shengzhong Instrument Co., Ltd., Qindao, China). When the desorption equilibrium was reached, the suspension was centrifuged, thus determining the concentrations of the desorbed Mn(II) solution. The final Mn(II) concentrations in solution were analyzed. The desorption capacity of the Mn(II)-loaded-nano@LC/MT was calculated according to the following Equations (6).

6 $${q}_{t,2}=\frac{{C}_{t,2}\times {V}_{2}}{{m}_{2}}$$

where *q*_*t*,2_ (mg/g) refers to the desorption amount at time *t* (min). *C*_*t*,2_ (mg/L) refers to the concentration of Mn(II) in the desorbed solution at time *t* (min). *V*_2_ (mL) refers to the total volume of solution in desorption. *m*_2_ (g) refers to the mass of the adsorbent after adsorption of Mn(II).

Repeated batch experiments were performed to examine the reusability of nano@LC/MT for Mn(II). After the desorption equilibrium was completed, the suspension was separated from the adsorbent by centrifugation at 6000 rpm for 10 min, washed with deionized water to remove the remaining acid, and vacuum-dried in an oven (DZF-6210, Shanghai Yiheng Scientific Instrument Co., Ltd. Shanghai) at 85 °C for the next adsorption of Mn(II). The adsorption and desorption capacities of Mn(II) were determined and analyzed. The consecutive adsorption/desorption processes were performed five times.
